# Parliament and Publications

**Published:** 1910-10-15

**Authors:** 


### Parliament and Publications.

### THE COST OF POOR-LAW MEDICAL RELIEF.

The total amount shown to have been expended by poor-law authorities in England and Wales, on medical relief last year was £896,711, of which £634,434 represents the cost in the case of indoor paupers, and £262,277 the cost in the case of outdoor paupers. The above-mentioned sum includes the sums expended on the salaries and extra medical fees of poor law medical officers; on the salaries or other remuneration of dispensers, nurses, and other officials employed in the treatment or care of the sick; and on medical and surgical appliances, drugs, etc. The sum spent on medical relief in the preceding year was £855,049. The total numbers of men, women and children receiving outdoor medical relief only on January 1, 1910, were respectively 6,156, 5,890, and 5,002. "Thirty-ninth Annual Report of the Local Government Board," Part I. (Cd. 5260). Price 1s. 3d.

### THE SALE OF PROPRIETARY MEDICINES.

That there has been a slight decrease in the sale of proprietary medicines is indicated by the fact that the receipts from medicine-stamp duty during the year ended March 31, 1910, was £2,375 less than in the previous year. According to the first annual report of the Commissioners of Customs and Excise, which has just been issued, the revenue from medicine stamp duty was £313,114 during the year under review, as compared with £315,489 in the previous year, and £334,142 in the year before that. The sale of proprietary remedies appears to have reached the high-water mark in the last-mentioned year—that is to say, in the year ending March 31, 1908. During the last ten years the revenue derived from the sale of medicine stamps reached no less a sum than £3,206,036. It may be interesting to examine what this means. The duty on a medicine the selling value of which is one shilling or less is three halfpence; it follows, therefore, that during the last ten years the British public has purchased the equivalent of 512,965,760 packages of secret or proprietary remedies of the selling value of one shilling, for which it has paid £25,648,288. These figures are not strictly accurate, because all the medicines sold are not priced at one shilling; but they give a fairly approximate idea of the vast sums squandered on quack remedies. The number of persons in Great Britain licensed to sell so-called "patent medicines" is 42,413, of whom 39,113 are in England and 3,300 in Scotland. "First Report of the Commissioners of His Majesty's Customs and Excise" (Cd. 5302). Price 9d.

